# Molecular Basis of Redox Signaling

**DOI:** 10.1155/2019/6414975

**Published:** 2019-05-05

**Authors:** Maria Clara Franco, Maria C. Carreras, Luciana Hannibal

**Affiliations:** ^1^Department of Biochemistry and Biophysics, College of Science, Oregon State University, Corvallis, OR, USA; ^2^Departamento de Bioquímica Clínica, Facultad de Farmacia y Bioquímica, Universidad de Buenos Aires, Buenos Aires, Argentina; ^3^University Medical Center Freiburg, Freiburg im Breisgau, Germany

Oxidants are produced in physiological and pathological conditions. The production of reactive nitrogen and oxygen species (RNS and ROS, respectively) can lead to vastly different cellular outcomes depending on their subcellular location, half-life, reactivity, gradients, and the antioxidant defenses. While oxidative stress caused by general oxidative damage is often nonspecific and linked to cell death by necrosis, at lower concentrations, ROS and RNS can act as second messengers regulating redox-sensitive signaling pathways, which elicit very specific cellular responses [1, 2]. Redox signaling is an intrinsic, tightly regulated component of cell metabolism, controlling cell growth, differentiation, and death. The interplay between the production of oxidants and the antioxidant defenses is highly regulated to maintain cellular redox homeostasis [3, 4]; thus, its dysregulation underlies many pathological conditions, including cancer, neurodegeneration, and cardiovascular and metabolic diseases. This special issue is focused on redox signaling in pathology and developments in redox-based therapies.

In this issue, H. Pérez et al. examined the molecular basis of the role of p66Shc in brain mitochondrial dysfunction, showing a connection with ROS/RNS production, Sirt 3 activity modulation, and mitochondrial dynamics and biogenesis. The modulation of p66shc activity could be a candidate for therapeutic intervention for longer lifespan or higher quality of life. In an article exploring the stimulatory role of nitric oxide (NO) on protein synthesis in the muscle, R. Wang et al. show that protein synthesis is regulated in a NO-dependent manner in differentiated C2C12 myoblasts by the mTOR/p70S6K pathway, highlighting the potential clinical applications of targeting the production of NO in muscle metabolism. Also, by a thorough examination of heart function and metabolites in a mouse Nrf2 knock-out model, R. Erkens et al. show that inhibition of NO synthesis at the onset of ischemia (I) and during early reperfusion (R) worsened myocardial damage and systolic dysfunction in Nrf2-deficient animals. The study reveals that eNOS upregulation under conditions of compromised antioxidant capacity may afford cardioprotection against I/R. K. Xu et al. discuss tryptophan and its metabolites as a source of antioxidant defense in the placenta. The authors summarize data on mechanisms that involve tryptophan and 3-hydroxyanthranilic acid, a metabolite of the tryptophan-kynurenine pathway, as initiators of signaling events that activate Nrf2 in the placenta, stimulating the expression of antioxidant proteins and proteins responsible for the biosynthesis of low molecular weight antioxidants.

In relation to cancer, S. V. Kostyuk et al. examined the penetration of easily oxidizable GC-rich DNA fragments (GC-DNA) into breast cancer cells and their potential for therapeutic development. Results from the study indicate that GC-DNA provided in the cell culture medium interacts with the cell surface, induces NOX4 expression, and undergoes oxidation by augmentation of reactive oxygen production. Thus, the incorporation and expression of cell-free DNA in cancer cells may harbor utility for therapeutic development.

Additional articles in this special issue address fundamental aspects of redox signaling in physiological conditions. P. V. Avdonin et al. uncovered that H_2_O_2_ synergizes with calcium-mobilizing agonists of the 5-hydroxytryptamine receptors 5-HT1B and 5-HT2B through the induction of endogenous oxidative stress in human umbilical vein endothelial cells and suggested that H_2_O_2_ potentiation of agonist-induced calcium signaling in endothelial cells may contribute to vasorelaxation. In another study by C. Urbainsky et al., the authors performed a comprehensive analysis of the substrates and functions of the oxidoreductase nucleoredoxin (Nrx) in neuronal cells. Their results suggest that Nrx may be involved in the redox regulation of cell morphology and metabolism.

Four review articles cover current knowledge of the role of oxidants and redox signaling in pathology. S. Boukhenouna et al. discuss the biological role of reactive oxygen species in the progression of chronic obstructive pulmonary disease (COPD) and cellular mechanisms to repair oxidative damage. A primary factor in the development of COPD is exposure to cigarette smoke that induces oxidative stress and epithelial cell damage. Cigarette smoke is the leading risk factor of lung injury and pulmonary emphysema. Sustained oxidative damage to biomolecules forces chronic upregulation of antioxidant and other cytoprotective responses. Under the high oxidative stress conditions documented in patients with COPD, defense systems can reach exhaustion failing to halt further disease deterioration. E. Richard et al. summarize the current knowledge on the pathophysiological role of reactive oxygen species and redox imbalance in inborn errors of metabolism (IEM), with a focus on selected branched-chain amino acid disorders, organic acidurias, and homocystinuria. The authors also cover the source of ROS in these IEMs and evaluate the efficacy of antioxidant therapies and mitochondria-targeted approaches in these diseases. Also, A. Giuffrè and J. B. Vicente present a comprehensive review of the biochemistry of hydrogen sulfide and its intricate interactions with other gasotransmitters. The work covers hydrogen sulfide occurrence in biological fluids and tissues, biogenesis and catabolism by specialized enzymes, partition in intra- and extracellular compartments, signaling, abnormal homeostasis in cardiovascular disease, neurodegenerative disorders and cancer, and reactivity with NO and CO. The review by R. Manoharan et al. describes the role of serine-threonine kinase receptor-associated protein (STRAP) in the regulation of cell proliferation and death in normal and cancer cells. While STRAP interacts with many redox-sensitive proteins to promote cell survival and proliferation in both normal and cancer cells, under conditions that affect the redox balance, STRAP may also induce cell death. This dual role of STRAP in cancer cells may uncover novel therapeutic options for cancer treatment.

Therapeutic strategies aimed at restoring redox homeostasis have been pursued for many years, albeit with very limited success. In this special issue, N. E. Buglak et al. present a critical review of the literature, evaluating successful and unsuccessful approaches, including patient selection, dose, and delivery route. The authors also focus on the development of local targeted delivery of antioxidants as a promising therapeutic approach to address local redox dysfunction. For example, J. Paprocki et al. study how different markers of the oxidant-antioxidant equilibrium are modulated after repeated stimulation with hyperbaric oxygen (HBO), a therapy associated with increased production of reactive oxygen species used to treat sudden sensorineural hearing loss. Another example is the use of cold physical plasma (CAP), a therapeutic strategy that generates reactive oxygen and nitrogen species, used for chronic and acute wound treatment. In this issue, A. Schmidt et al. show that CAP activates a complex cellular response and discuss the functional consequences of the clinical use of plasmas for wound treatment.

The lack of tools to isolate specific oxidative modifications has hindered our understanding of the functional consequences of such modifications for many years. Here, J. J. Porter and R. A. Mehl summarize current knowledge of oxidative posttranslational protein modifications and the development of new tools through genetic code expansion, for the genetically encoded, site-specific incorporation of amino acids carrying oxidative modifications into proteins. These tools enable the accurate determination of the role of a particular posttranslational modification at unique positions in a protein of choice.

In summary, this special issue highlights the importance of understanding the molecular basis of redox signaling and the need for targeted therapies that have so far remained a major challenge in the field.
